# Moyamoya Disease Causing Stroke in the Setting of Cocaine Use and Uncontrolled Hypertension Due to Primary Hyperaldosteronism

**DOI:** 10.7759/cureus.51578

**Published:** 2024-01-03

**Authors:** Nathan DeRon, Francis Fischer, Tara Norris

**Affiliations:** 1 Internal Medicine, Methodist Health System, Dallas, USA

**Keywords:** puff of smoke, cocaine use disorder, moyamoya syndrome, moyamoya disease, cocaine, hyperaldosteronism, stroke, moyamoya

## Abstract

Moyamoya disease is a cerebrovascular disease characterized by stenosis of large intracranial arteries and the development of smaller collateral vessels. Moyamoya may cause strokes and stroke-like symptoms in young patients. It has also been linked to autoimmune diseases and neuropsychiatric conditions. We present a case of moyamoya disease in a young patient with concomitant hyperaldosteronism, uncontrolled hypertension, and cocaine use disorder, along with features of antisocial personality disorder. This is a unique presentation of an underlying neurological disease causing psychiatric features exacerbated by cocaine use, and it describes a rare clinical presentation that physicians should consider in patients with moyamoya disease.

## Introduction

Moyamoya disease is a cerebrovascular disease characterized by stenosis of large intracranial arteries and the development of smaller collateral vessels [1]. Moyamoya may cause strokes and stroke-like symptoms in young patients [2]. It has also been linked to autoimmune diseases and neuropsychiatric conditions [3-5]. Moyamoya disease was first described in Japan in the 1950s [6] and receives its iconic name from neuroimaging showing copious small collateral intracranial vessels resembling a “puff of smoke” such as from a cigarette [7]. The prevalence of moyamoya disease is difficult to define as the disease is exceedingly rare and not always identified; however, estimates of prevalence are between 0.1 and 0.5 cases per 100,000 patients [7].

The diagnosis of moyamoya disease is based on clinical presentation and neuroimaging findings of bilateral distal ICA stenosis and the development of small-caliber collateral vessels [8,9]. Moyamoya disease is a distinct entity in which the disease is identified independent of associated conditions and is usually considered only in bilateral ICA stenosis as opposed to moyamoya syndrome, which is typically in the setting of other conditions, such as autoimmune disorders or sickle cell anemia and may present unilaterally [10].

We present the case of a young patient newly diagnosed with moyamoya disease and discuss common presentations and management of this disease entity with an emphasis on building clinician education and awareness.

## Case presentation

The patient is a 35-year-old African American female, with a past medical history of hypertension who presented to the emergency department with acute right upper extremity weakness and right facial droop. She reported a two-day history of right arm weakness and progressive right facial droop, which did not resolve with observation. She then reported calling emergency medical services who arrived on the scene and reported the patient’s blood pressure as 240 mmHg/160 mmHg. The patient also reported associated increasing confusion for which she required family at her bedside to assist with her medical history. The patient denied any previous history of similar episodes. The patient and family denied any known family medical history of similar symptoms. The patient did admit to smoking one pack of cigarettes per day for approximately 17 years and near-daily marijuana use.

The patient was found to be tachycardic to 120 beats per minute and hypertensive to 197/123 mmHg on physical exam. The patient was alert but profoundly confused and only oriented to name, which deviated significantly from her neurologic baseline, which was fully functional and independent. A right facial droop was noted. The right upper extremity was noted to have 3/5 strength and intact sensation. All other physical exam findings were within normal limits. Initial laboratory data are presented in Table 1.

**Table 1:** Summary of inpatient laboratory findings. BUN = Blood Urea Nitrogen; TSH = Thyroid Stimulating Hormone; HDL = High-Density Lipoprotein; LDL = Low-Density Lipoprotein

Lab	Value	Reference
Sodium	135 mmol/L	135-145 mmol/L
Potassium	2.6 mmol/L	3.4-5.1 mmol/L
Chloride	105 mmol/L	95-106 mmol/L
Carbon Dioxide	20 mmol/L	22-31 mmol/L
BUN	9 mg/dL	10-25 mg/dL
Creatinine	0.91 mg/dL	0.7-1.4 mg/dL
Glucose	122 mg/dL	70-110 mg/dL
TSH	2.39 ulU/mL	0.5-5.0 uIU/mL
Aldosterone	8.5 ng/dL	3.1-35.4 ng/dL
Renin Activity	0.3 ng/mL/hr	0.2-1.6 ng/mL/hr
Hemoglobin A1c	6.0%	< 5.7%
Triglycerides	81 mg/dL	< 150 mg/dL
Cholesterol	104 mg/dL	< 200 mg/dL
HDL	27 mg/dL	35-80 mg/dL
LDL	44 mg/dL	< 190 mg/dL
Urine Drug Screen	Cocaine Positive	Negative

**Figure 1:**
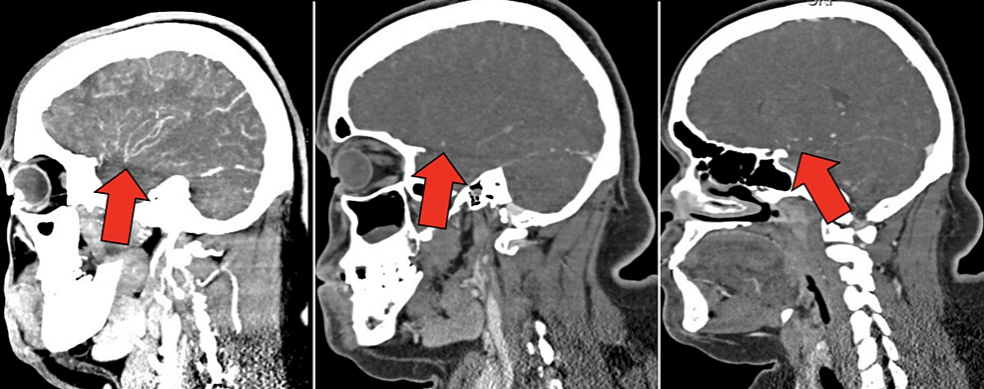
Sagittal CTA images illustrating right MCA luminal irregularities (left), narrow caliber left MCA (middle), and narrow caliber left terminal ICA (right). CTA = Computed Tomography Angiography; MCA = Middle Cerebral Artery; ICA = Internal Carotid Artery

**Figure 2:**
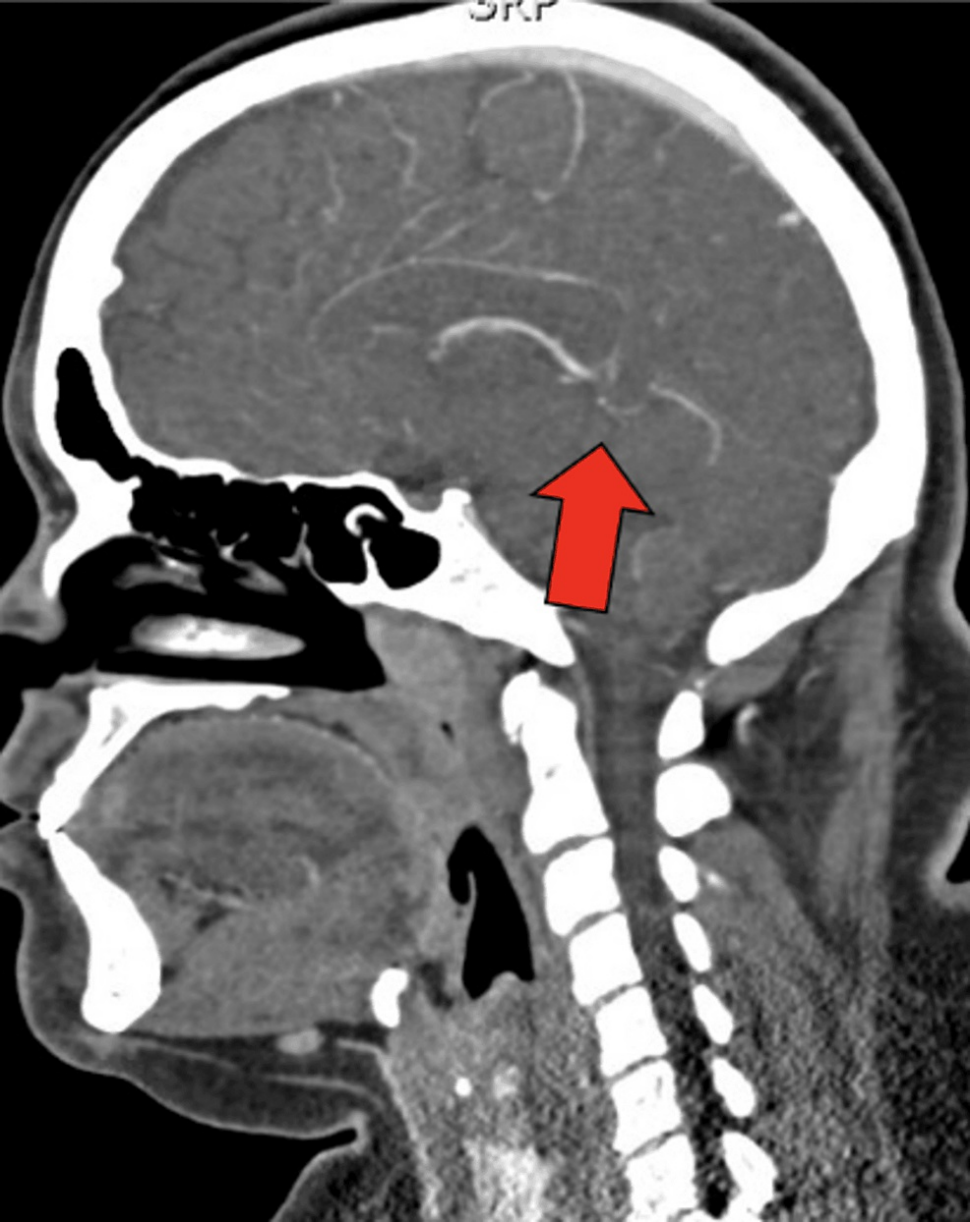
Sagittal CTA image demonstrating right P3 segmental stenosis.

**Figure 3:**
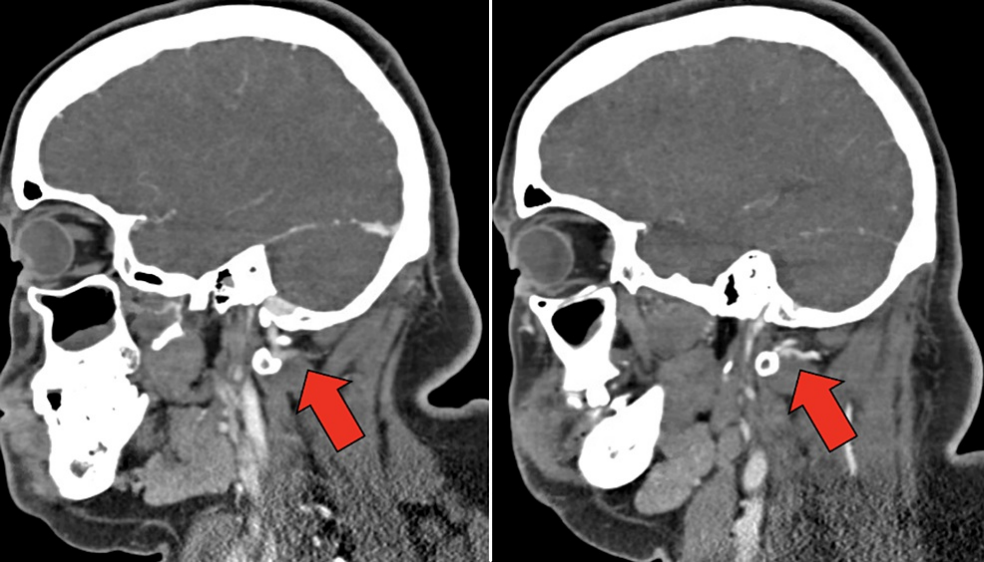
Narrow caliber left vertebral artery (left) and narrow caliber right vertebral artery (right).

**Figure 4:**
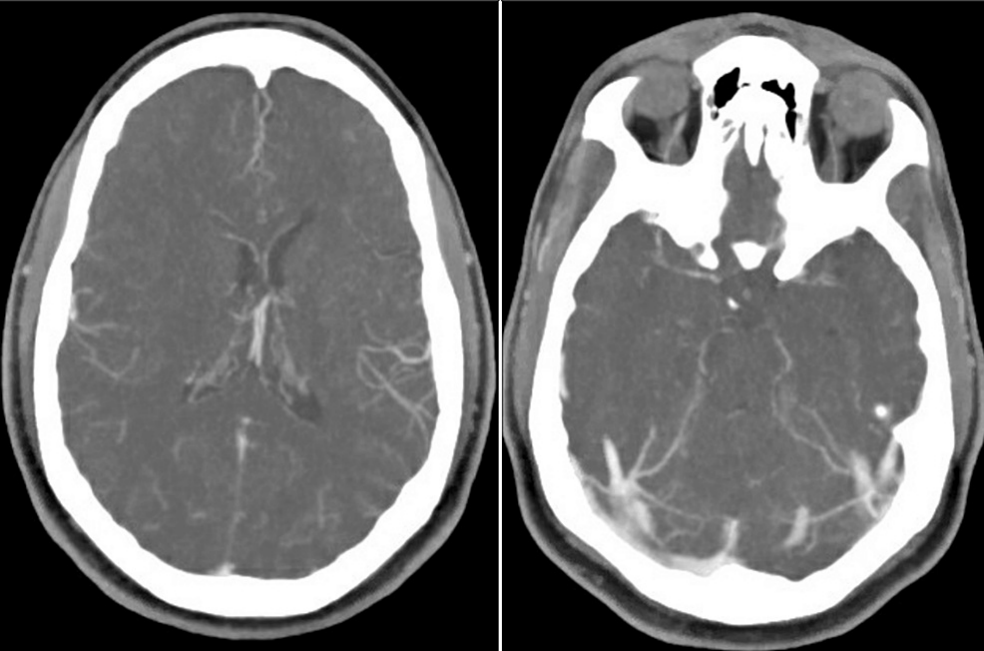
Axial CTA images showing numerous small caliber collateral vessels within the brain. CTA = Computed Tomography Angiography

Electrocardiography revealed sinus tachycardia with voltage criteria for left ventricular hypertrophy. Computed tomography (CT) of the head revealed no acute abnormalities, such as hemorrhage. CT angiogram (CTA) of the head and neck revealed bilateral narrowing of the internal carotid arteries (ICAs) with associated luminal irregularities (Figure 1). There was high-grade stenosis with possible occlusive disease along the left carotid terminus. The left M1 segment showed diminutive vessel caliber with luminal irregularities throughout the left M1, M2, and M3 segments. The right internal carotid artery also showed a small caliber terminus. The bilateral anterior cerebral arteries revealed segmental stenosis and luminal irregularities. The right posterior cerebral artery showed a short segment of high-grade stenosis along the P3 segment (Figure 2). The bilateral vertebral arteries were noted to have a small caliber (Figure 3). Numerous collaterals were noted to be scattered throughout the brain parenchyma (Figure 4). The interpretation of the CTA was challenging due to artifacts from beam-hardening and venous contamination.

**Figure 5:**
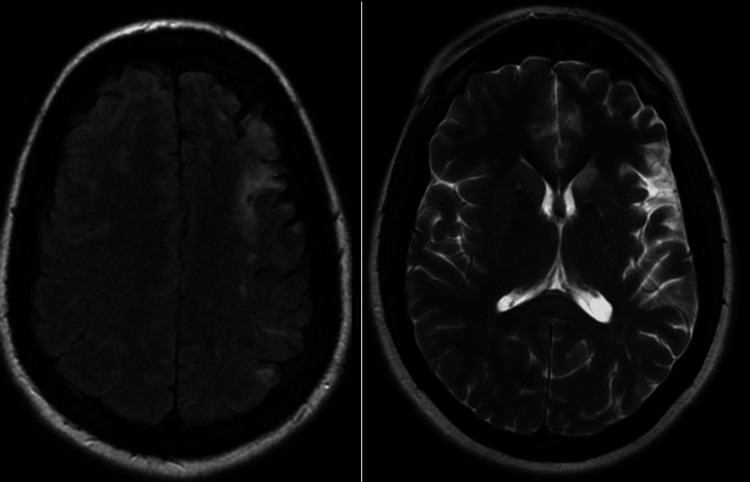
T2 FLAIR demonstrating a large ischemic lesion in the left MCA and ACA territories (left). T2 PROPELLER redemonstrating the left frontoparietal and the left caudate head lesions.

**Figure 6:**
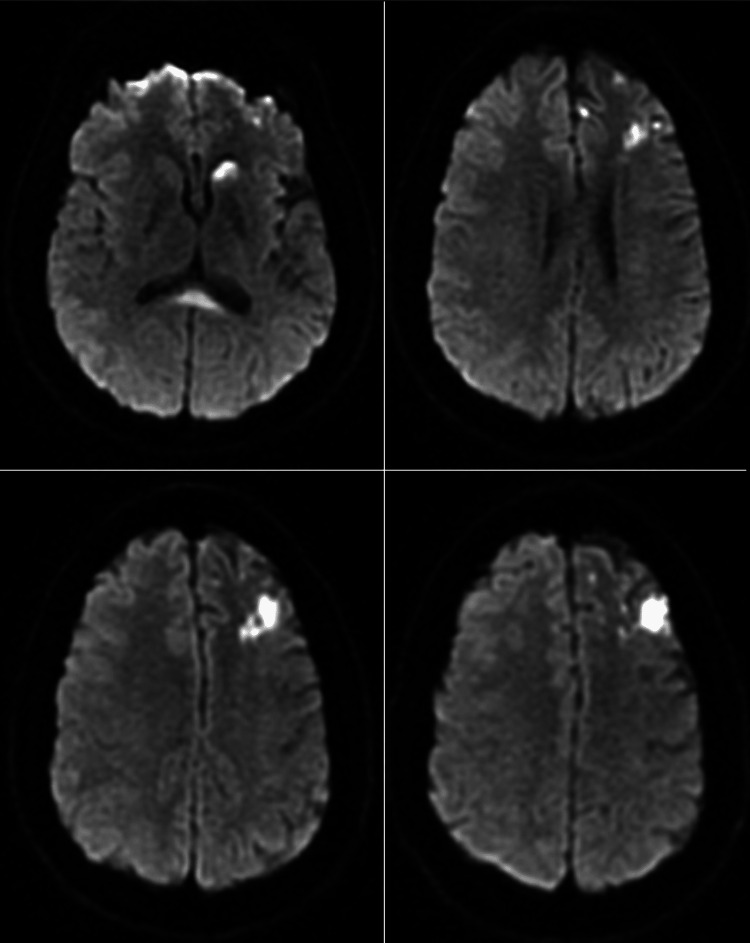
Axial DWI images revealing increased Brownian motion and illustrating ischemic changes in the left caudate head and splenium callosum (top left) and left frontal and parietal lobes (top right, bottom left, bottom right). DWI = Diffusion-Weighted Imaging

The patient was admitted to the medicine service for an ongoing stroke workup. Magnetic resonance imaging (MRI) of the brain was obtained for further evaluation of stroke (Figures 5, 6). The MRI revealed nodular ischemic changes along the left frontal and parietal white matter and left caudate head. The midline callosal splenium also exhibited diffusion restriction. There was coexisting left frontal lobe encephalomalacia and gliosis with chronic ischemic changes in the left periventricular and subcortical tissues.

Given the imaging findings of recent ischemic changes along the left frontal lobe, left parietal lobe, and left caudate head, the patient was diagnosed with an acute ischemic stroke secondary to newly diagnosed moyamoya disease in the setting of recent cocaine use and uncontrolled hypertension. Neurosurgery was consulted, and the patient was initially planned for a cerebral angiogram for percutaneous intervention planning. The patient was also started on low-dose aspirin and high-intensity statin therapy. The patient's blood pressure was carefully titrated to avoid relative hypotension. The patient improved over the next two days of hospitalization with a slow return of right upper extremity strength and a decrease in right facial droop. As the patient recovered, she became increasingly agitated and exhibited antisocial personality traits such as significant abrasiveness with her care team and impulsiveness when discussing management options. Upon chart review, these personality features were documented up to approximately 10 years prior to the current hospitalization. There was no fatigue, increased appetite, or psychomotor depression to indicate cocaine withdrawal; therefore, these characteristics were attributed to a long-standing personality disorder.

The patient’s blood pressure was noted to be difficult to control. A bilateral renal ultrasound was performed but did not reveal any renal artery stenosis or renal perfusion abnormality. Upon chart review from outside facilities, the patient was noted to be significantly hypertensive as early as 23 years old. Therefore, the decision was made to evaluate the patient for secondary hypertension. The plasma aldosterone-renin activity ratio was noted to be abnormally elevated at 28.2, indicating the likelihood of primary hyperaldosteronism. The patient also continued to exhibit persistent hypokalemia despite aggressive replacement therapy and adequate magnesium levels consistent with hyperaldosteronism.

Ultimately, the patient became increasingly confrontational and requested discharge after the resolution of her stroke symptoms. The patient refused cerebral angiography and further endocrinology workup. The patient was placed on low-dose aspirin, high-intensity statin, and angiotensin receptor blocker therapies and discharged with instructions to follow up outpatient in one week. The patient was unfortunately lost to follow-up.

## Discussion

Cerebral vasculature is best visualized with cerebral angiography; however, vessel imaging with CTA or magnetic resonance angiography is a suitable non-invasive alternative that can also confirm the diagnosis. Some data even advocate for high-resolution vessel wall imaging in young patients presenting with stroke symptoms to expedite management [11]. On MRI, patients often have sequelae of previous ischemic changes similar to the patient in this case. Structural alteration patterns of subcortical gray matter and atrophy of the hippocampus and amygdala can also be seen in moyamoya and may suggest cognitive or affective impairment [12]. The patient in this case exhibited characteristics of antisocial personality disorder potentially caused or exacerbated by underlying moyamoya disease.

There are several genetic factors that have been linked to moyamoya disease. In younger patients, a 15q11.2 deletion may be present, and patients can exhibit symptoms such as epilepsy, autism spectrum disorder, and delays in psychomotor development [13]. This clinical finding is consistent with the patient in this case who may have suffered delayed cognition or neurodevelopment secondary to moyamoya.

The constellation of stroke symptoms, psychiatric disorders, and cognitive delay in a relatively young patient should prompt clinicians to include moyamoya in the differential diagnosis [14]. However, there are additional facets of the clinical presentation that should be considered as well. The prevalence of psychiatric disorders in moyamoya patients has been shown to be higher than the general population and most often presents as mood and anxiety disorders [4]. This may also serve as a bridge to alcohol and illicit drug abuse such as cocaine use disorder. Cases have been reported of moyamoya patients who are also cocaine users experiencing sudden and premature death [15]. The pathophysiology of this clinical consequence is obvious as cocaine is a potent cerebral vasoconstrictor [16], which only exacerbates cerebral hypoperfusion in patients with moyamoya disease.

Moyamoya is associated with other disease entities, including autoimmune and prothrombotic disorders, which are found at significantly higher rates in patients with moyamoya compared to the general population [17]. It has also been shown to be more prevalent in East Asians and Caucasians [9] likely due to a common mutation in the RNF213 gene [18], whereas moyamoya in the African American population is more infrequent [19,20]. African American patients are also less likely to experience ischemic strokes with moyamoya [21]. Moyamoya has also been associated with renovascular hypertension [22], possibly leading to secondary hypertension.

Management of moyamoya typically includes revascularization if the primary locations of stenosis are easily accessible by percutaneous catheterized intervention. Some studies have shown excellent results after revascularization from stenting or indirect bypass [17], including a reduction in the incidence of recurrent stroke [23,24]. Surgical revascularization has also shown efficacy in certain subsets of the disease population, including African American and Caucasian patients [25]. However, there is mixed data on this topic as revascularization may be achieved, but neurocognitive performance may not improve after intervention [26]. Medical management with long-term low-dose aspirin is also part of the standard of care and has been shown to improve survival in moyamoya patients [27].

## Conclusions

This case of moyamoya disease represents a rare clinical presentation in an even rarer demographic causing an ischemic stroke. Moyamoya disease can be challenging for clinicians to both diagnose and manage given its rarity and ability to closely mimic other causes of acute ischemic stroke. The association of moyamoya with psychiatric disorders may further exacerbate the clinical complexity and potentially interfere with management as in this case. It is important to emphasize to clinicians that moyamoya should be on the differential in young stroke patients, and early imaging and revascularization can alleviate symptoms and possibly mitigate future stroke risk.
